# A qualitative RT-PCR assay for the specific identification of the SARS-CoV-2 B.1.1.529 (Omicron) Variant of Concern

**DOI:** 10.1016/j.jcv.2022.105191

**Published:** 2022-07

**Authors:** Philippe Corbisier, Mauro Petrillo, Antonio Marchini, Maddalena Querci, Gerhard Buttinger, Meriem Bekliz, Katja Spiess, Charlotta Polacek, Anders Fomsgaard, Guy Van den Eede

**Affiliations:** a European Commission, Joint Research Centre (JRC), Geel 2400, Belgium; b Seidor Italy SRL, Milano, Italy; c European Commission, Joint Research Centre (JRC), Ispra, Italy; d Geneva Centre for Emerging Viral Diseases, Department of Microbiology and Molecular Medicine, University of Geneva, University Hospital Geneva (HUG), Switzerland; e Virus Research & Development lab, Department of Virus & Microbiologic Special Diagnostics, Statens Serum Institut, Denmark

**Keywords:** SARS-CoV-2, Omicron, B.1.1.529, RT-PCR, Specific identification

## Abstract

**Objectives:**

The aim of this study was to develop a RT-PCR assay for the specific detection of the SARS-CoV-2 Omicron Variant of Concern (VOC) as a rapid alternative to sequencing.

**Methods:**

A RT-PCR was designed *in silico* and then validated using characterised clinical samples containing Omicron (both BA.1 and BA.2 lineages) and the Omicron synthetic RNA genome. As negative controls, SARS-CoV-2 positive clinical samples collected in May 2020, and synthetic RNA genomes of the isolate Wuhan Hu-1 and of the Alpha (B.1.1.7), Beta (B.1.351), Gamma (P.1), Kappa (B.1.617.1), Iota (B.1.526), Epsilon (B.1.429) and Delta (B.1.617.2) SARS-CoV-2 VOC were used.

**Results:**

Experiments performed using as templates the synthetic RNA genomes demonstrate the high specificity of the PCR-method for the SARS-CoV-2 Omicron. Despite the synthetic RNAs were used at high copy numbers, specific signal was mainly detected with the Omicron synthetic genome. Only a non-specific late signal was detected using the Alpha variant genome, but these results were considered negligible as Alpha VOC has been replaced by the Delta and it is not circulating anymore in the world. Using our method, we confirmed the presence of Omicron on clinical samples containing this variant but not of other SARS-CoV-2 lineages. The method is highly sensitive and can detect up to 1 cp of the Omicron virus per µl.

**Conclusions:**

The method presented here, in combination with other methods in use for detection of SARS-CoV-2, can be used for an early identification of Omicron.

## Highlights

• A one-step RT-PCR assay for the specific detection of SARS-CoV-2 Omicron.
• The assay was evaluated *in silico*, and validaded on synthetic RNA genomes and clinical samples.
• The analytical sensitivity and specificity of the assay allow the fast detection of SARS-CoV-2 Omicron even at low concentration level.
• In contrast to other RT-PCR assays based on the deletion 69-70, this assay is able to detect the Omicron lineage BA.1 but also the currently prevalent BA.2 lineage.
• The assay represents an advantageous alternative to sequencing or to the *S* gene target failure assays.

## 1 Introduction

A novel SARS-CoV-2 variant (Pango lineage B.1.1.529) was first reported to the World Health Organization (WHO) (https://www.who.int/news/item/26-11-2021-classification-of-omicron-(B.1.1.529)-sars-cov-2-variant-of-concern) from the Republic of South Africa on 24 November 2021 and was classified two days later as a VOC with the name “Omicron” (for a simplified reference) [1]. First identified in countries in the southern part of Africa, the variant has quickly appeared worldwide. Omicron has already become the dominant variant in Europe by replacing the Delta variant, and is replacing other variants in other continents.

Omicron is characterised by more than 50 nucleic acid changes (including deletions). Most of these mutations reside in the S gene encoding for the spike glycoprotein [2]. The spike plays a central role in the entry of the virus into human cells through interaction with the human ACE2 receptor [3,4], and is the main antigenic component of mRNA- and viral vector-based vaccines [5], [6], [7], [8]. Consistent evidence indicates that by acquiring these mutations, the Omicron variant infects the cells more efficiently, replicates faster and spreads at superior rate in comparison to other previously emerged variants [2,9]. These mutations may also render certain vaccines against SARS-CoV-2 less effective in preventing infection [10], [11], [12], [13], [14].

After less than one month (19 December 2021) from its first reporting, 11,615 SARS-CoV-2 genomic sequences had been deposited in GISAID [15], 10,755 of them were flagged as “complete” and with less than 5% undetermined bases. After two months from its first reporting, according to the Pango nomenclature, four Pango lineages were defined as Omicron B.1.1.529, BA.1, BA.2 and BA.3. By screening  the S gene of all Omicron-flagged complete sequences, we identified unique and Omicron-specific sequences of B.1.1.529, BA.1 and BA.2 (which together include more than 99% of Omicron sequences) to be used for the development of a novel reverse transcriptase polymerase chain reaction (RT-PCR) based method for the specific identification of  the Omicron.

## 2 Materials and methods

### 2.1 *In silico* design of the RT-PCR method

SARS-CoV-2 genomic sequences deposited in GISAID, flagged as “complete” and with less than 5% undetermined bases have been downloaded and analysed with Nextclade [16] (Supplementary Material S1). Only mutations present in at least 80% of the analysed sequences were retained to define a set of commonly shared mutations. The *S* gene, in which most of these mutations occur, was manually inspected to look for genomic regions not longer than 250 bp and with at least 6 mutations. The National Center for Biotechnology Information (NCBI) Reference Sequence NC_045512.2 was used as reference [17]. A region (NC_045512.2:22950-23150) was identified with these features and fed into *Primer3Plus* [18] to design *in silico* primers and probes methods (program run with pre-loaded qPCR settings). A candidate method, specific for the genomic region NC_045512.2:22991-23128, was selected. We call this method OMicron METhod (OmMet) [19]. OmMet has been tested *in silico* by using the *thermonucleotideBLAST* software [20] with the following parameters: *-e 20 -E 20 -l 200* on the selected set of SARS-CoV-2 genomic sequences. A set of Pango lineages [1] consensus sequences was obtained by running an in-house developed script that parses data retrieved from the Broad Institute COVID CG [21] application programming interface (used *consensus_threshold of 0.9*). This set was used to check *in silico* the OmMet specificity. Sequence alignments were produced by using MAFFT online service [22] and alignments’ representations were obtained by running the *showseq* tool of EMBOSS package [23].

### 2.2 Samples analysed

The samples used in this study are listed in Table 2. The five clinical samples assigned as Omicron-positive were from nasopharyngeal swabs from COVID-19 positive patients at the University Hospital of Geneva (HUG). Primary diagnosis of SARS-CoV-2 infection was made by RT-PCR using the Cobas 6800 SARS-CoV-2 RT-PCR assay (Roche, Basel, Switzerland). Furthermore, viral RNA was extracted using the NucliSENS easyMAG extraction kit (BioMérieux, CH) protocol followed by screening for *S* gene target failure (SGTF) with the TaqPath COVID19 assay (ThermoFisher). Sample with SGTF were further analysed by partial Sanger sequencing of the spike region [24]. Confirmation of the Omicron variant was done by full genome sequencing fusing Illumina NovaSeq and the sequence from 3 samples submitted to GISAID. The viral load expressed in cp/µL was measured by RT-PCR using the Twist Bioscience Control 48 as calibrant. Three additional clinical samples - two SARS-CoV-2 positive (Wuhan Hu-1 lineage) and one negative nasopharyngeal swabs- were kindly, provided in viral transport media by Dr Elke Wollants (University of Leuven, KUL) in March 2020. Viral RNAs was extracted from these samples using the QIAamp Viral RNA Kit (QIAGEN) according to manufacturer's instructions and used as negative controls in the RT-PCR.

Additionally, clinical samples of lineages BA.1 and BA.2 were tested in the Virus Research & Development lab (Denmark), to verify whether OmMet performs efficiently with both lineages. Samples were collected and tested SARS-CoV-2 positive from community testing facilities (Test Centre Denmark) and BioBank Denmark, which form part of the Danish national surveillance program [25]. Viral genomes from throat swabs collected in 1x PBS were isolated using the MagNa Pure 96 nucleic acid extraction system (Roche) with 100 µL elution. The identities of the lineages BA.1 and BA.2 were confirmed by Whole Genome Sequencing (WGS) generated by the Danish COVID-19 Genome Consortium (DCGC) [26] and consensus genomes were deposited in GISAID (gisaid.org).

*In vitro* transcript synthetic RNAs covering the full viral genomes of the SARS-CoV-2 Wuhan Hu-1 lineage and indicated variants (Table 2) were purchased from Twist Bioscience (South San Francisco, CA, USA) and diluted 10 time in Nuclease free-water supplemented with carrier RNA before use at a calculated concentration of 5 × 10^5^ cp/RT-PCR reaction.

### 2.3 RT-PCR assays

The sequences of the oligo's and TaqMan® probe are provided in Table 1. The TaqMan® probe for the OmMet assay was labelled at the 5′-end with the reporter molecule 6-carboxyfluorescein (FAM) and with the QSY-7 succinimidyl ester quencher [QSY™ from Applied Biosystems, CA) at the 3′-end. The QSY is designed for optimal sensitivity and performance in multiplex qPCR experiments, but it can be replaced by dark quencher probes, such as Black Hole Quencher (BHQ™) probes without changing probe sequences. OmMet targets a region (138 bp) located in the *S* gene (region NC_045512.2:22991-23128). The 3’ mismatches on the forward primer and on the probe were chosen to increase the specificity of the assay. The assay targeting the E gene (E-Sarbeco) was used as unspecific control assay to determine the presence of the SARS-CoV-2 virus and estimate the viral load [27].

**Table 1:** Primers and TaqMan® probe sequences and relative concentrations used for the E gene and OmMet simplex PCR assays. Omicron-specific nucleic acid base changes are highlighted in red.

Target	Code	Oligo	Final concentration [nM]	Ref.
E gene	E-F1	5’-ACAGGTACGTTAATAGTTAATAGCGT-3’	400	[27]
	E-R2	5’-ATATTGCAGCAGTACGCACACA-3’	400	
	E-P1	5’-FAM-ACACTAGCCATCCTTACTGCGCTTCG-BBQ-3’	200	
S gene	Omt-F	5’-AACAAACCTTGTAATGGTGTTGC-3’	900	This paper
	Omt-R	5’-TGCTGGTGCATGTAGAAGTTC-3’	900	
	Omt-P	5’-FAM-GATCATATAGTTTCCGACCCACTTATGGTGTTGGTC-QSY-3’	300	

The reaction mixtures for the RT-PCR experiments were prepared as follows: for each 25 µL reaction, 5 µL of the extracted RNA were added to 20 µL of the reaction mix containing 10.75 µL of H_2_0 (RNAse free), 1 µL of primers and probe mix (forward and reverse primers at final concentration of 900 nM and FAM-QSY labelled probe at a final concentration of 300 nM) and 6.25 µL of MasterMix (TaqPath 1-step RT-qPCR MasterMix 4 x, ThermoFisher Scientific). The PCR programme consists of uracil-DNA glycosylase incubation (2 min at 25°C), reverse transcription (15 min at 50°C), TaqMan® activation (2 min at 95°C) and 45 cycles of amplification (3 s at 95°C followed by 30 s at 62°C). All RT-PCR reactions were performed in a calibrated QuantStudio 7 Flex Real-Time PCR System (Applied Biosystems, BE); the raw data were analysed with the QuantStudio software (version 1.3) using an automatic threshold setting.

The OmMet assay was also performed using the same primers (Table 1) but with the Luna® Universal Probe One-Step RT-qPCR Kit and the Luna WarmStart® RT Enzyme Mix (New England Biolabs) at two different annealing temperatures (58°C and 62°C) to verify the robustness of the assay on BA.1 and BA.2 Omicron samples.

## 3 Results

### 3.1 *In silico* validation

**Fig. 1:**
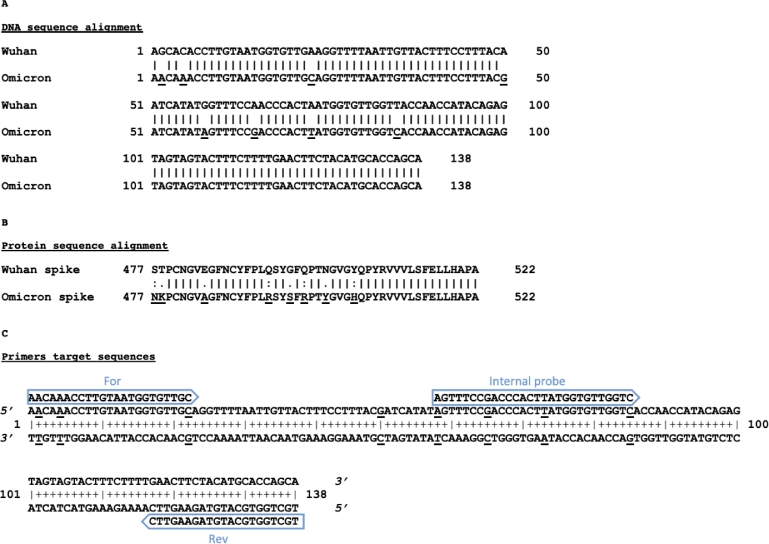
OmMet target amplicon. A. The part of the Omicron genome (S gene) amplified by OmMet was aligned with the corresponding region of the original reference sequence of the SARS-CoV-2 Wuhan strain (NC_045512.2:22991-23128); bases corresponding to the mutations present in Omicron, and found in this area, are underlined. **B.** Protein sequence alignment. The region amplified by OmMet was translated into protein and aligned with the corresponding Spike protein reference sequence (aa 477-522) of the Wuhan strain. The mutated amino acids are underlined. **C.** 138 bps amplicon (and its reverse complement) target of OmMet: OmMet primers and probe sequences are blue-boxed; mutations present in Omicron are underlined. For: forward primer, Rev: reverse primer

The initial design of OmMet was carried out by inspecting 305 Omicron genomic sequences deposited between 16/11/2021 and 02/12/2021 in GISAID flagged as complete, and with  less  than  5% undetermined bases (Supplementary Material S1). The overview of the OmMet target amplicon (within the S gene) compared to the SARS-CoV-2 homologous sequence (Wuhan strain, NCBI Reference Sequence NC_045512.2, nucleotides: 22991-23128) is shown in Fig. 1 A. Eight point mutations were found in this region of the Omicron genome in comparison to the reference sequence These mutations lead to amino acid changes as found by align the corresponding fragments of the Omicron and reference Wuhan strain Spike proteins (amino acids 477-522) (Fig. 1 B). Primers and probe were designed considering these unique features present within the Omicron genome (Fig.1 C).

OmMet was tested *in silico* and found with perfect matching on the selected set of Omicron sequences retrieved from GISAID (Supplementary Material S1). Additionally, OmMet was tested *in silico* on the consensus sequences of all known Pango lineages [1]. In all the analysed sequences, differences were found in the regions of annealing of both the forward and reverse primers, and of the hybridisation oligo (Alignment in Supplementary Material S2). These *in silico* results underline the OmMet's ability to detect the Omicron sequences with very high specificity.

**Fig. 2:**
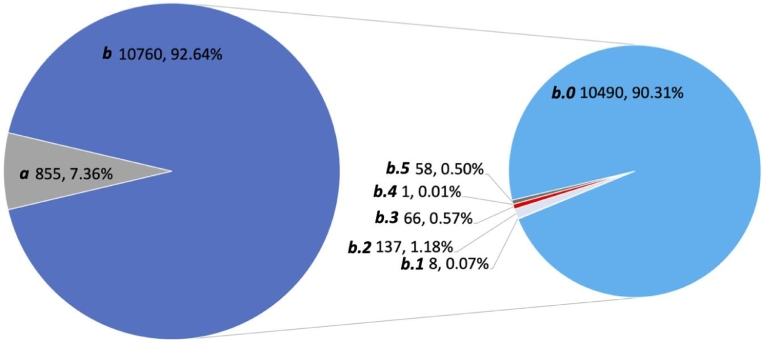
*In silico* prediction of OmMet ability to specifically detect Omicron.   A total of 11615 GISAID deposited as Omicron sequences between 16/11/2021 and 18/12/2021 were analysed for their ability to be recognised and amplified by OmMet. The group (a) is composed of sequences that are not predicted to be amplified by OmMet due to stretches of undetermined bases (as N) covering primers or oligo target regions of the analysed sequences. The group (b) is instead composed of sequences predicted to be amplified by OmMet. The subgroups include sequences identical to the primers and probe (b.0); sequences with undetermined bases outside the primers and probe targets (b.1), sequences with undetermined bases inside the primers and probe targets (b.2), sequences with mismatch(es) inside the primers and probe targets (b.3), sequences with a frameshift (b.4), and sequences with mismatches which make them very similar to to the WUHAN sequence (b.5). For each group and subgroups, the number of sequences analysed is provided, together with their corresponding percentage calculated on the total number of sequences analysed.

*In silico* simulation predicts that the OmMet is highly specific for the detection of Omicron (Fig. 2). About 7% of the 11,615 analysed sequence data set deposited in GISAID and flagged as Omicron have long stretches of N in one or more of the target regions (group a), and can therefore not be detected *in silico*. Among the remaining ones (group b) more than 97.5% (sum of subgroups b.0 and b.1) have target regions perfectly matching with primers and probes of the OmMet method. Only 66 sequences (subgroup b.3) have not-N mismatches on these target regions, thus representing potential false negatives. Interestingly, 58 sequences (subgroup b.5) flagged as Omicron do not show the typical Omicron nucleic acid changes, and consequently they represent false positives, i.e. sequences wrongly assigned to Omicron.

### 3.2 Analytical specificity of the Omicron assay

We evaluated the specificity of the OmMet RT-PCR assay on RNA extracted from five characterized Omicron positive patients and two SARS-CoV-2 positive samples collected at the onset of the pandemic in March 2020 (containing the Wuhan Hu-1 prototype). Together with the OmMet, we also analyzed these samples with the E-Sarbeco method, which is widely used for the detection of SARS-CoV-2 [27]. The presence of Omicron was confirmed in Omicron positive samples by both OmMet and Sarbeco methods with similar Ct values (Table 2). On the contrary, the clinical samples containing the Wuhan Hu-1 were detected only by the E-Sarbeco method, providing evidence of the high specificity of OmMet for the Omicron variant. One Omicron positive sample was serially diluted (1 x, 10 x, 100 x, 1000 x). Despite the dilutions, OmMet was still able to easily detect the virus. A nasopharyngeal sample tested positive with the E-Sarbeco assay with a very low Ct of 12.5 was negative with the Omicron assay, confirming the high specificity of the method.

**Table 2:** Threshold values (Ct) reported for the clinical samples and Quality Control samples tested by the E-Sarbeco assay and the OmMet RT-PCR specific assay. Values reported with a standard deviation are average of triplicates. Threshold values reported as “> 45” indicate that the target is not detected, values reported as “>” gives the lowest Ct values measured with some replicates > 45. The threshold for both assays was set manually at 0.1 to compare the Ct values. NTC: no template control. *: measured by HUG, #: measured by KUL, nt: not tested.

Sample Origin and GISAID ID	Viral load estimated in (cp/reaction)	SARS-CoV-2 lineage	E - Sarbeco (Ct value)	S gene OmMet (Ct value)
HUG(hCoV-19/Switzerland/VD-HUG-36220991/2021)	2.1 × 10^4^	Omicron B1.1.529 BA.1	23.9*	21.2
	6.6 × 10^3^		n.t.	23.9
	5.7 × 10^2^		n.t.	27.5
	6.1 × 10^1^		n.t.	30.8
HUG(hCoV-19/Switzerland/VD-HUG-36221084/2021)	1.2 × 10^4^	Omicron B1.1.529 BA.1	21.8*	23
HUG(hCoV-19/Switzerland/VD-HUG-36220960/2021)	1.7 × 10^3^	Omicron B1.1.529 BA.1	19.7*	24.4
HUG – sample 3	4.4 × 10^4^	Omicron B1.1.529 BA.1	22.2*	21.1
HUG – sample 5	2.7 × 10^3^	Omicron B1.1.529 BA.1	n.t.	25.2
KUL – sample 48	5.1 × 10^5^	Wuhan- Hu-1	15.9^#^	> 45
KUL – sample 63	8.0 × 10^3^	Wuhan- Hu-1	26.7^#^	> 45
KUL – sample 425	0	Negative	> 45^#^	> 45
Twist Bioscience - control 2	5 × 10^5^	Wuhan- Hu-1	18.7 ± 0.01	> 42.9
Twist Bioscience - control 14	5 × 10^5^	Alpha B.1.17	19.5 ± 0.04	33.1 ± 0.2
Twist Bioscience - control 16	5 × 10^5^	Beta B.1.351	19.3 ± 0.02	39.2 ± 0.1
Twist Bioscience - control 17	5 × 10^5^	Gamma P1	19.3 ± 0.01	39.9 ± 1.4
Twist Bioscience - control 23	5 × 10^5^	Delta B.1.617.2	20.0 ± 0.01	> 43.8
Twist Bioscience - control 21	5 × 10^5^	Epsilon B1.429	20.6 ± 0.03	> 45
Twist Bioscience - control 19	5 × 10^5^	Iota B.1	18.9 ± 0.07	> 45
Twist Bioscience - control 18	5 × 10^5^	Kappa B.1.617.1	19.7 ± 0.01	> 45
Twist Bioscience - control 48	5 × 10^5^	Omicron B1.1.529 BA.1	19.2 ± 0.03	19.6 ± 0.03
NTC	0	-	> 45	> 45

The specificity of the assay was also verified by testing synthetic RNA covering the full genome of the Wuhan Hu-1, the Alpha, Beta, Delta, Gamma, Kappa, Iota, Epsilon and Omicron at a high concentration (calculated to be around 5 × 10^5^ copies per PCR reaction) (as listed in Table 2).

Similar Ct values were obtained using the synthetic Omicron genome (B1.1.529 BA.1) as template with both the E-Sarbeco and the OmMet assays (19.2 ± 0.03 and 19.6 ± 0.03 respectively) indicating that both assays perform equally well with Omicron.

As expected, OmMet failed to detect the Kappa, Epsilon and Iota variants and detected all other variants at an extremely low sensitivity with signal revealed only after 39 cycles or later, except for the Alpha variant. For the Alpha variant, a signal was detected after 33 cycles, showing that OmMet is at least 16,000 times less efficient in detecting this variant than the E-Sarbeco method for which a Ct value of 19.5 was measured at the same concentration level. It is important to point-out that the Alpha variant is not circulating anymore in the EU/EEA following the emergence of Delta variant.

**Fig. 3:**
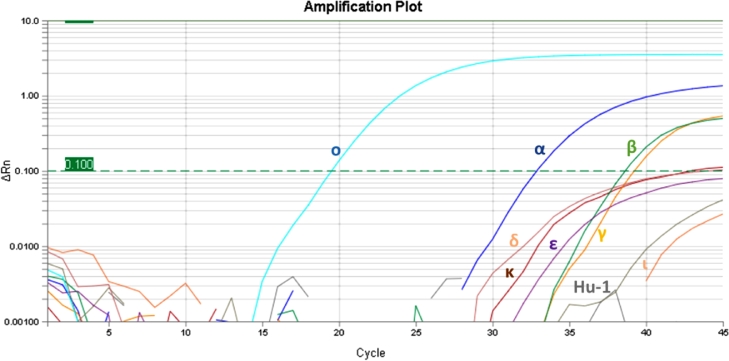
Amplification plot for the positive control material (sample 48 - labelled ο) and the negative control synthetic materials (labelled with their Greek name). Hu-1: Wuhan Hu-1 prototype.

Typical amplification plots are illustrated in Fig. 2, the late parasite amplifications observed for the Alpha (and to a lesser extension for the Gamma variant) show a potential limitation of the RT-PCR, which despite the presence of 8 mismatches among the oligos and their targets, still provides a weak signal after many PCR cycles (plateau phase). However, potential false positives with the Alpha variant are not likely to occur as this “de-escalating” variant has been replaced in Europe by the Delta one, which gives a very week and late signal even when present at very high concentration in the RT-PCR assay (Fig. 3 and Table 2).

After the design of OmMet, two novel lineages of Omicron (BA.2 and BA.3) have been described. These two novel Omicron lineages are lacking the mutation A23040G in the S-region which is present in BA.1. As the OmMet was *in silico* designed using as a template the Omicron BA.1 sequences, the TaqMan probe developed contains this specific mutation (at position 9) (Fig. 1C and Supplementary Material S1). Therefore, there is a potential risk that OmMet would perform less efficiently with BA.2 and BA.3 that do not share this mutation. We decided to challenge OmMet with samples containing the Omicron BA.2 lineage. This part of the study was carried out at the Virus Research & Development laboratory (Statens, Denmark), also for testing the applicability of the method in another laboratory and its robustness when using different reagents and different annealing temperatures for the RT-PCR. In particular, in addition to the recommended temperature of 62 °C, the annealing temperature of 58 °C was tested.

The results showed that both BA.1 and BA.2 are detectable with very similar Ct values at both annealing temperatures, although, a superior specificity against the Hu-1, the Alpha and Delta variants was achieved with the recommended annealing temperature of 62 °C rather than 58 °C (Table 3).

**Table 3:** Threshold values (Ct) reported on Danish clinical samples containing the Omicron BA.1 and BA.2 lineages and measured at two different annealing temperatures.

Sample ID	S gene OmMet (Ct value)	
	Annealing Temp. 62 °C	Annealing Temp. 58 °C
(10000 x) SARS-Cov2 wt	> 45	42.79
Delta B.1.617.2 - 1000 x fort	> 45	34.46
TWIST control 1×10^-^^3^ Alfa (British)	44.46	41.66
Omicron BA.1 patient sample 1	29.68	29.83
patient sample 2	25.66	25.69
patient sample 3	25.49	26.09
patient sample 4	31.13	31.35
patient sample 5	24.43	24.70
Omicron BA.2 patient sample 1	31.92	31.39
patient sample 2	30.22	30.06
patient sample 3	28.74	28.55
patient sample 4	30.92	30.96
patient sample 5	31.61	31.88
Sample TCD (negative SARS-CoV-2)	> 45	> 45
NTC (H_2_O)	> 45	> 45

As expected, the RT-PCR assay is performing with similar efficiency when using different MasterMixes and different concentrations of the primers.

### 3.3 Analytical sensitivity of the Omicron assay

At the time of this study, omicron positive clinical samples available at the HUG and SSI had relatively high viral loads with Ct values ranging from 21.1 to 31.92 representing a limitation when assessing the analytical sensitivity. To estimate the analytical sensitivity of the method, extracted RNA from the sample VD-HUG-36220991v (Table 1) was diluted 10, 100 and 1000 times and the respective Ct values for the S-gene target recorded. For the highest dilution, corresponding to 61 copies per reaction, a Ct value of 30.8 was recorded which suggests an even lower detection limit, knowing that signal up to Ct values of approximatively 37 can be detected.

**Fig. 4:**
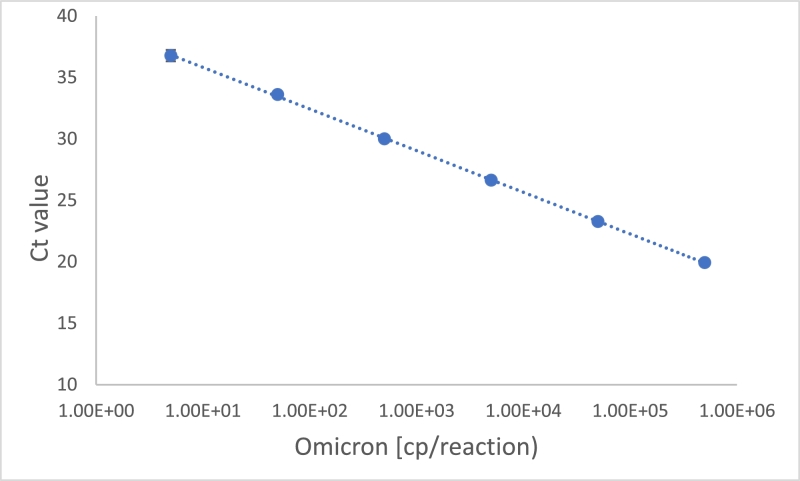
Linearity of the RT-Assay tested with the IVT Omicron positive control. Each point represents the average of 3 replicates. Error bars are provided as standard deviation.

The analytical limit of detection and linearity of the signal were therefore further studied by recording the Ct values of diluted synthetic Omicron genome in the PCR reaction (Fig. 4). Concentration up to 1 cp/µL was detected by OmMet and the signal was linear between 5 to 50000 cp/reaction showing a sufficient analytical sensitivity of the method and a PCR efficiency of 97.3 %.

## 4 Discussion

The epidemic potential of the new SARS-CoV-2 Omicron variant is of considerable concern due to its apparent higher transmissibility and the likely negative effect that many of the mutations it features might have upon natural or vaccine-induced immunity and on our ability to detect the virus.

Currently, public health officials mostly rely on the so-called S gene target failure (SGTF) phenomenon to quickly identify Omicron in patient samples [24]. SGTF occurs in commercial assay designed to detect sequences from three SARS-CoV-2 genes – *S, N* and *ORF1ab*. Due to the large number of mutations in the *S* gene of Omicron, assays give positive signal for *N* and *ORF1ab* presence, for the new variant, but negative for *S* and such a test result is interpreted as from a sample containing Omicron. Although much more rapid than sequencing identification, SGTF suffers from lower specificity. For example, the *S* gene of many rare SARS-CoV-2 variants features a genetic sequence that is not recognised by the assays. In addition, swab samples with lower virus concentration may fail to detect the *S target*. Furthermore, the *S* gene of some actual Omicron lineages can in fact be detected by the tests, resulting in a lack of discrimination with the SGTF approach. The method we propose has a very high accuracy, typical for the sequencing approaches, while allowing much shorter turn over times needed for public health response and decision making.

SARS-CoV-2 continues to evolve with its spreading. The same Omicron already evolved into several lineages and sub-lineages. It is not clear whether these novel emerging viruses have growth advantages in comparison to the initially prevalent BA.1. Despite being designed on BA.1, we verified that OmMet is able to detect also the lineages BA.2 (and most likely BA.3, BA.4 and BA.5 lineages), that is the prevalent lineage in Europe at the time of writing (end of April 2022).

We provide also evidence of the robustness of OmMet. The method performs equally well with different masterMixes (TaqPath 1-step RT-qPCR MasterMix and the Luna® Universal Probe One-Step RT-qPCR Kit) and it is expected to work with reagents from other providers, too. The RT-PCR assay was tested using primers and probe at 900 nM and 300 nM, and at a lower concentration of 450 nM and 214 nM, respectively, without decrease in performances (data not shown). For a high specificity towards Omicron, we recommend to use the protocol with an annealing temperature of 62°C.

This method is simple to be settled up and implemented in any biotech/clinical/environmental laboratory that is able to carry out PCR assays, and should aid in globally monitoring the spread of the Omicron variant. The method could be combined with other RT-PCR methods in use for the detection of SARS-CoV-2 (e.g. the E-Sarbeco method [27]) to discriminate between Omicron and other SARS-CoV-2 variants. In this regard, it can also be used as part of a monitoring approach aimed to reveal emerging of novel potential VOCs. In fact, Omicron being currently the predominant variant circulating in the world, negative results obtained with OmMet, but positive to other SARS-CoV-2 methods which recognize conserved elements of the SARS-CoV-2 genome, may indicate that other SARS-CoV-2 variants with mutations in the S gene target region recognized by OmMet primers and probe are emerging.

To the best of our knowledge, when this paper was published as a preprint [19,28], OmMet was the first single one-step RT-PCR assay reported for the specific detection of Omicron. Since then, two other PCR-based assays have been developed for the detection of Omicron in clinical and environmental samples [29,30]. These methods take advantage of other unique features present in the S gene of Omicron (e.g. typical deletions or insertions) and have been described to be highly specific and sensitive for Omicron. However, according to our *in silico* amplification, the del69-70 based assays as described in [29,30], should not be able to detect the lineage Omicron BA.2, which is currently (end of April 2022) prevalent, as it is missing that particular deletion.

OmMet is suitable to be combined in a multiplex approach with other methods to closely track the evolution of the virus in surveillance programs. In this regard, recent results performed in our laboratory (not shown, but in preparation) and in national reference laboratories [31] show that OmMet equally performed well with RNAs extracted from wastewater samples. Alternatively, Bloemen et al. have recently described a method that combines PCR amplification of the S gene (corresponding to the region coding for amino acids 360 to 588 of the Omicron Spike) with Sanger sequencing [32] to detect VOCs . That method in comparison to the others may unequivocally ascertain the presence of VOCs (including Omicron) in given samples. However, as it uses a sequencing step, it is necessarily more laborious and time consuming in surveillance programs.

In conclusion, this study describes the design of primers and probe to identify the Omicron variant by a RT-PCR. The method has the necessary specificity and sensitivity to discriminate the Omicron variant from other SARS-CoV-2 variants.

## 5 Conclusions

The epidemic potential of the new SARS-CoV-2 Omicron variant is of considerable concern and even if whole Genome Sequencing (WGS), or at least complete or partial S gene sequencing remains the best method for characterising a specific variant, we provide here a diagnostic RT-PCR method that can be used for an early identification of Omicron. The analytical sensitivity and specificity were demonstrated *in silico*, on a (limited) number of clinical containing both BA.1 and BA.2, and on control samples. We feel its sharing with the scientific community is critical and invite control laboratories to use that method to confirm the clinical sensitivity and specificity of this assay on Omicron positive samples.

## Authors’ contributions

PC, AM, MP, MQ and GB designed the study. MB provided the RNA from Omicron positive swap specimens. GB, KS and CP extracted RNA from Omicron negative swap specimens and performed RT-PCR experiments. PC, AM and GB analysed data. MP designed the OmMet method. PC, AM and MP wrote the manuscript. PC, AM, MP and MQ reviewed the manuscript. GVdE and AF supervised the study. All authors discussed the results and commented on the final manuscript.

## Declaration

The scientific output expressed does not imply a policy position of the European Commission. Neither the European Commission nor any person acting on behalf of the Commission is responsible for the use that might be made of this publication.

## Declaration of Competing Interest

The authors declare no competing interests.

## Appendix Supplementary materials

Image, application 1

Image, application 2

## Footnotes

Supplementary material associated with this article can be found, in the online version, at doi:10.1016/j.jcv.2022.105191.
